# Knowledge-infused Learning for Entity Prediction in Driving Scenes

**DOI:** 10.3389/fdata.2021.759110

**Published:** 2021-11-25

**Authors:** Ruwan Wickramarachchi, Cory Henson, Amit Sheth

**Affiliations:** ^1^ Artificial Intelligence Institute, University of South Carolina, Columbia, SC, United States; ^2^ Bosch Research and Technology Center, Pittsburgh, PA, United States

**Keywords:** neuro-symbolic computing, knowledge-infused learning, knowledge graph embeddings, autonomous driving, scene understanding, entity prediction

## Abstract

Scene understanding is a key technical challenge within the autonomous driving domain. It requires a deep semantic understanding of the entities and relations found within complex physical and social environments that is both accurate and complete. In practice, this can be accomplished by representing entities in a scene and their relations as a knowledge graph (KG). This scene knowledge graph may then be utilized for the task of entity prediction, leading to improved scene understanding. In this paper, we will define and formalize this problem as Knowledge-based Entity Prediction (KEP). KEP aims to improve scene understanding by predicting potentially unrecognized entities by leveraging heterogeneous, high-level semantic knowledge of driving scenes. An innovative neuro-symbolic solution for KEP is presented, based on knowledge-infused learning, which 1) introduces a dataset agnostic ontology to describe driving scenes, 2) uses an expressive, holistic representation of scenes with knowledge graphs, and 3) proposes an effective, non-standard mapping of the KEP problem to the problem of link prediction (LP) using knowledge-graph embeddings (KGE). Using real, complex and high-quality data from urban driving scenes, we demonstrate its effectiveness by showing that the missing entities may be predicted with high precision (0.87 Hits@1) while significantly outperforming the non-semantic/rule-based baselines.

## 1 Introduction

Knowledge graphs are capable of representing meaningful relations between entities in the world; and they are now being developed, at large scale, for various applications and uses. One such application gaining in prominence is knowledge-infused learning, a technique for integrating—or infusing—knowledge into machine learning models (Valiant, 2006; Sheth et al., 2019; Garcez and Lamb, 2020). This infusion of knowledge has been shown to improve the predictive capabilities of machine learning/deep learning models. Examples include 1) recommendations (Chen et al., 2017), 2) visual and textual concept learning (Mao et al., 2018) and 3) question answering (Ma et al., 2019). Additionally, knowledge-infused learning has displayed great potential for improving the intepretability and explainability of ML/DL predictions (Gaur et al., 2020; Palmonari and Minervini, 2020; Tiddi et al., 2020).

For these reasons, knowledge-infused learning holds much promise for helping to meet the complex technical challenges of scene understanding that’s inherent in autonomous driving (AD). Scene understanding typically involves processing a multitude of data streams from an array of sensors including cameras, LIDAR and RADAR. This data is then used to detect, recognize and track the objects and events in a scene. While ML/DL techniques have been successful in solving these challenges (Grigorescu et al., 2020), they may lack the ability to fully utilize the interdependence of entities and semantic relations within a scene. We will demonstrate that knowledge-infused learning can exploit such information to further improve our ability to understand driving scenes.

We will consider one scene understanding challenge in particular: *knowledge-based entity prediction* (hereafter referred to as *KEP*). We define KEP as *the task of predicting the inclusion of potentially unrecognized entities in a scene, given the current and background knowledge of the scene represented as a knowledge graph.* We hypothesize that, **a knowledge-infused learning approach—with an expressive KG representation of scenes—would provide rich, high-level semantic cues needed to predict the unrecognized entities within a given scene.** For example, consider the scenario of an autonomous vehicle driving through a residential neighborhood on a Saturday afternoon. Its perception module detects and recognizes a ball bouncing on the road. What is the probability that a child is nearby, perhaps chasing after the ball? This prediction task requires knowledge of the scene that’s out-of-scope for traditional computer vision techniques. More specifically, it requires an understanding of the semantic relations between the various aspects of a scene; e.g. that the ball is a preferred toy of children, that children often live and play in residential neighborhoods.

**FIGURE 1:**
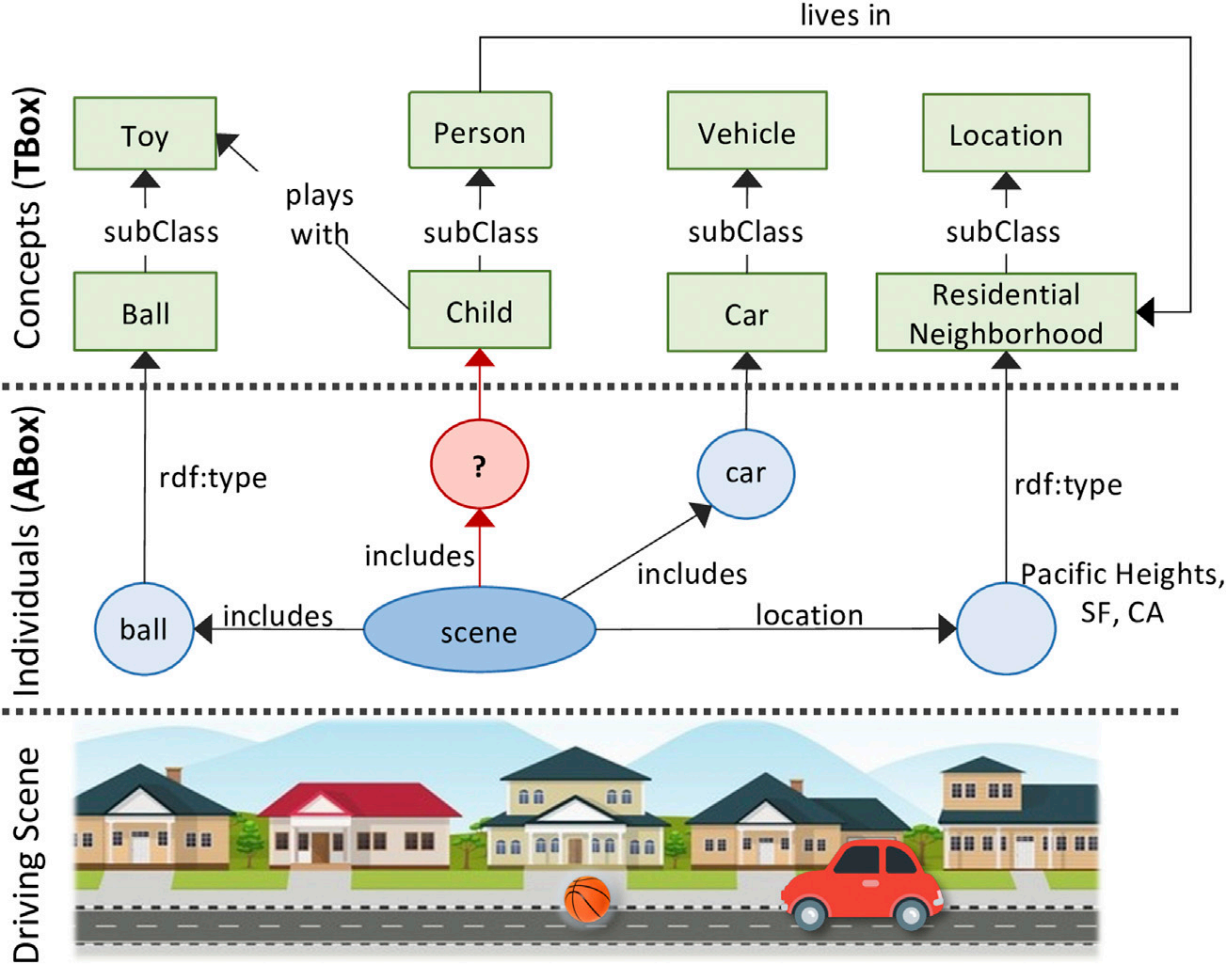
Example scene KG with potentially missing entity.

Portraying such relational knowledge of a scene requires a representation that is expressive and holistic. In computer vision, a scene is often represented as a set of labeled bounding boxes drawn around the objects detected within a frame. However, as shown above, driving scenes are more complex than just a set of recognized objects. In this regard, we agree with Ramanishka et al. (2018) who argue that parsing visual scenes into a set of semantic categories is only the first step toward a rich and high-level scene understanding. In addition, scene data is often multi-modal, distributed, and originating from multiple sources. This necessitates the integration of scene information into a unified, holistic representation. A knowledge graph of scenes satisfies both of these criteria: the ability to 1) integrate heterogeneous information, and 2) represent rich semantic relations (Figure 1 for example).

In this paper, we propose a knowledge-infused learning approach for scene entity prediction. This approach begins with exploring several autonomous driving datasets (Geiger et al., 2013; Caesar et al., 2019; Scale AI, 2020) and identifying the various spatial, temporal and perceptual components that comprise a scene. These components are then semantically defined and structured within an ontology. Scene data from AD datasets are transformed into a KG, conformant to the ontology, which represents a wide and varied selection of driving situations. Next, the KG is translated into a knowledge graph embedding (KGE) (Wang et al., 2017), which encodes the KG in a low-dimensional, latent feature vector representation. Three popular embedding algorithms are used for this purpose: TransE (Bordes et al., 2013), HolE (Nickel et al., 2016), and ConvKB (Nguyen D. Q. et al., 2018). Finally, the KGE is used to perform scene entity prediction. This is accomplished by mapping KEP to the well-known problem of link prediction (LP), commonly discussed in the KG completion literature. This mapping is not straightforward, however, given the insight that KEP may be more accurately formalized as a *path prediction* problem. This challenge is ultimately overcome by applying an inventive process of path reification. The performance of KEP is evaluated, analyzed, and discussed. The evaluation shows that HolE significantly outperforms the other embedding algorithms for the KEP task, achieving a peak precision of 0.87 for Hits@1 with one of the high-quality datasets. In addition, the evaluation covers several investigations into the effects of KG structure and external knowledge on the KEP task.

It is important to note that the focus of this paper is to define a general *knowledge-infused learning approach for KEP* and demonstrate its capabilities with real driving scene data. Therefore, the specific combination of datasets, algorithms, or hyperparameter settings described in this paper are not optimized for peak KEP performance. Such details are included in order to demonstrate proof-of-concept.

The primary contributions of this paper include:

1. Introducing the Knowledge-based Entity Prediction (KEP) task and proposing an innovative knowledge-infused learning approach.
2. Mapping KEP to the well-known problem of KG link prediction, showing it’s limitations and how they can be overcome through a process of path reification.
3. Developing a dataset agnostic ontology to describe driving scenes.

The rest of the paper proceeds as follows: Section 2 discusses the related work. Details about the datasets, ontology, and knowledge graph are introduced in Section 3. The overall methodology and evaluation are presented in Section 4 and Section 5, respectively. Section 6 discusses additional investigations conducted on two incidental problems, and in Section 7 we provide an analysis and discussion on all evaluations. Finally, in Section 8, we wrap up with conclusions and future work.

## 2 Related Work

In this section we outline three important areas of related work, including: object detection and recognition, scene representation, and link prediction.

### 2.1 Object Detection and Recognition

**FIGURE 2:**
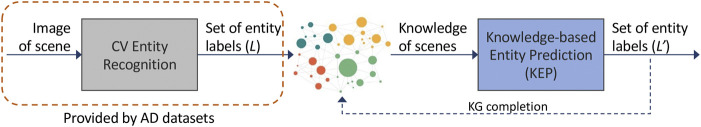
Knowledge-infused KEP as a post-processing step for computer vision entity prediction techniques, which takes a set of labels (L) as input and outputs a new set of labels (L’).

Object detection and recognition are key components in scene understanding. The objective is to detect objects and classify them into known semantic types. The input to this process could be either 2D images obtained from cameras or 3D point clouds generated by LIDAR. Note that, in each case, detected objects are recognized by the 2D/3D bounding-boxes drawn around them. Semantic segmentation, on the other hand, takes a more granular approach by assigning a semantic category to each pixel in an image. State-of-the-art DL architectures proposed for each of these methods can be found in (Grigorescu et al., 2020; Yurtsever et al., 2020). At a high-level, both object recognition and semantic segmentation produce a set of object label annotations for a given image or 3D point cloud, and such information is readily available in the AD datasets. KEP is distinct from these approaches in several ways. First the input to visual object detection methods may include raw images, video, or LIDAR point clouds. In contrast, KEP expects a set of semantic entity labels as the input. Also, object recognition intends to assign a semantic label to a detected object, while KEP aims at predicting *additional* semantic labels for the entire scene. These additional labels represent entities that should actually be in the scene but may have been missed by the object recognition methods. This could occur for various reasons, such as hardware limitations, occluded entities, poor field-of-view, or degraded visuals. Viewed in this manner, KEP can be seen as a post-processing step in a AD perception pipeline (Figure 2.)

### 2.2 Scene Representation

As described in the previous section, a scene can be represented as a set of labeled bounding boxes within 2D images or 3D point clouds. However, this representation may not be expressive enough to capture contextual information of a scene. Scene graph generation (SGG) (Xu et al., 2020) aims at solving this issue by representing the detected objects as nodes in a graph, with direct edges representing the relationships between these objects; for example, the location of an object (e.g. pedestrian *in front of* the ego-vehicle), part of an object (e.g. bicycle *has* wheels) or action of an object (e.g. car *overtakes* a truck). While this representation can locally represent scene objects and the basic relations among them, it lacks the ability to represent the global view of relations among detected objects/events, location, notions of time and easily integrate external knowledge (e.g. common-sense). Our proposed approach addresses these limitations by first representing global relational structure of driving scene components in an ontology, and then representing the detected objects and relevant metadata in a conformant knowledge graph.

To develop this ontology and KG, we used the ontology proposed by (Wickramarachchi et al., 2020) as a foundation and extended it in several ways. First, we unified and added a wide array of entity types (i.e. objects and event) encountered in multiple AD datasets. Second, we improved the support for location attributes such as Geometry (GPS coordinates) and Address (street address, points of interests, etc.). Third, we enriched the structuring of Event by categorizing the events a vehicle could encounter on the road into four main categories such as vehicular/pedestrian/weather/animal events. Several ontologies have been previously developed for use in the autonomous driving domain; e.g. for scene creation (Bagschik et al., 2018), and representing scenarios (de Gelder et al., 2020; Geng et al., 2017)). While DSO shares some commonalities with these ontologies, it is also distinct in two primary aspects: 1) While prior ontologies were often designed for ontological reasoning and inference, the purpose of DSO is simply to structure scene information that can be used to train KGEs. This led to a minimalist ontology design involving the necessary components of a scene (i.e. objects, events, spatio-temporal attributes). 2) DSO is also designed with the structure and composition of current (open) AD datasets in mind. This makes the process of generating a KG from a new AD dataset as straightforward as possible.

A few recent works in the area of AD have also explored the quality of KGEs based on intrinsic evaluation metrics (Wickramarachchi et al., 2020), synthetic data based KGs (Halilaj et al., 2021), and the integration of external knowledge with scene graphs (Suchan et al., 2020).

### 2.3 Link Prediction

Link prediction (LP) is a well-studied problem in KG literature that focuses on addressing the KG incompleteness issue. Formally, a KG is defined as G⊂N×R×N such that N=C∪I (Table 1 for list of notation used). The facts in G are represented as triples of the form ⟨*h*, *r*, *t*⟩ where h,t∈N and r∈R . LP aims to enrich G with new facts by predicting the missing links between existing nodes (Chen et al., 2020)—i.e. predicting head ⟨?, *r*, *t*⟩ or tail ⟨*h*, *r*, ?⟩. The techniques for LP can be categorized into two broad classes: symbolic and sub-symbolic (i.e. ML/DL). Symbolic LP techniques primarily exploit the observable features in a KG and use Rule Mining (Galárraga et al., 2015; Meilicke et al., 2018) and path ranking algorithms (Lao and Cohen, 2010; Lao et al., 2011) to infer the missing elements in any given triple. Recently, with the popularity of ML/DL algorithms, sub-symbolic-based LP methods have gained traction due to their superior performance. These techniques learn to predict links by first encoding KG nodes and relations as a latent vectorized representation in low-dimensional space; referred to as knowledge graph embeddings, or KGEs.

**TABLE 1:** List of notations used.

G	Knowledge graph
R	Set of all relations in G
N	Set of all nodes in G
C	Set of all class nodes in G
I	Set of all instance nodes in G
T	Set of triples; ⟨h,r,t⟩∈T
S	Set of Scene instance nodes
*E*	Set of Entity class nodes
	(i.e. Objects and Events)

#### Knowledge Graph Embeddings

There are now a wide variety of KGE-based techniques for LP. Researchers in this field have categorized these methods into meaningful classes based on their underlying algorithm (Wang et al., 2017; Rossi et al., 2021), including geometric, matrix factorization, and deep learning based methods. In geometric models, the LP objective is formulated such that relations between nodes are interpreted as spatial translations in a geometric space. The matrix/tensor decomposition models consider KG as a 3D adjacency matrix and the LP objective is modeled as a decomposition of a triple tensor into a bi-linear product, resulting in node vectors and relation vectors/matrices. Finally, in deep learning-based models, the LP task is modeled using neural networks and the node/relation embeddings are jointly learned with shared parameters of the layers. Beyond LP, the learned embedding space has been widely used to query about KGE facts for various downstream applications [e.g. (Celebi et al., 2019; Mohamed et al., 2020)]. Our proposed solution is also based on this approach, re-using the embedding space for KEP. For this task, we evaluate multiple ML-based LP techniques, one from each of these algorithm classes to examine which algorithm and class may work well. In addition to these three classes, there is a set of algorithms that leverage path information for LP. Such methods, including PtransE (Lin et al., 2015) and PConvKB (Ding et al., 2018), use local and/or global path information to improve prediction of direct links. KEP, however, focuses on predicting a path of n-hops (in our setting, *n* = 2).

## 3 Knowledge Graphs of Driving Scenes

Scene understanding relies on high-quality knowledge about a scene. Scene data are inherently multi-modal; with information generated from many sources, including cameras, LIDAR, RADAR, and various other sensors. To integrate such heterogeneous information into a single, unified semantic representation, we use knowledge graphs. To understand how this knowledge about scenes is created and represented, we will first describe the autonomous driving datasets in which the scene data originates. Next, a formal semantics of scenes is introduced, as defined by the Driving Scene Ontology (DSO). Finally, a constructed KG of scenes, conformant to DSO, is described.

### 3.1 Datasets

Over the past few years, the autonomous driving domain has seen an influx of good benchmark datasets; including PandaSet (Scale AI, 2020), NuScenes (Caesar et al., 2019), and KITTI (Geiger et al., 2013). These datasets typically contain the raw data generated by cameras, LIDAR, and RADAR sensors, along with high quality annotations. Two recent, large-scele AD datasets—PandaSet provided by Hesai and Scale, and NuScenes provided by Motional—are used to prototype and evaluate the knowledge-infused learning methods described in this paper. The first open-source dataset made available for both academic and commercial use, PandaSet includes complex driving scenarios—e.g. steep hills, construction, dense traffic and pedestrians, and a variety of times of day and lighting conditions—from two routes in Silicon Valley: 1) San Francisco; and 2) El Camino Real. It uses a full sensor suite of a self-driving-car; including a forward-facing LiDAR, a mechanical spinning LiDAR, six cameras and an on-board GPS/IMU.^1^ This dataset represents 103 driving sequences of 8 s each, composed of 48K camera images and 16K LIDAR sweeps. Each sequence is sampled into frames with a frequency of 10FPS. PandaSet provides a rich set of annotations with 28 cuboid labels (i.e. 3D bounding box) and 37 semantic segmentation labels. The semantic segmentation labels include more granular-level details such as smoke, car exhaust, vegetation, and driveable surface. When annotating objects, Pandaset uses the same unique identifier for an object when it appears across multiple frames.

NuScenes consists of 1,000 driving sequences of 20 s each, from routes in Boston and Singapore with heavy traffic and challenging driving situations. Each driving sequence is sampled into frames with a frequency of 2FPS. It has a rich diversity of scenes as they are from different continents, different weather types, different traffic patterns (left vs right-hand traffic), etc. It contains 23 3D bounding box labels as well as object-level attributes such as vehicular activity (e.g., parked/stopped/moving). The full dataset contains 1.4M camera images, 390K LIDAR sweeps, 1.4M RADAR sweeps and 1.4M bounding boxes across 40K frames. Note that, different from Pandaset, NuScenes uses a new set of identifiers to identify objects in each frame. Hence the same object will get a different identifier if it appears in a subsequent frame.

### 3.2 Driving Scene Ontology and Knowledge Graph

**FIGURE 3:**
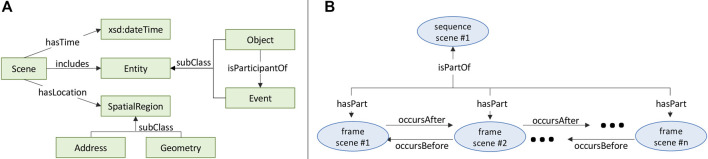
**(A)** Basic structure of a scene, **(B)** Two types of scene: sequence scene and frame scene.

The Driving Scene Ontology (DSO) provides a formal structure and semantics for representing information about scenes; formalized in OWL (McGuinness and Van Harmelen, 2004). A scene is defined as *an observable volume of space and time* (Henson et al., 2019). More colloquially, a scene typically refers to a situation in which objects may appear (e.g. vehicle) and events may occur (e.g. lane change maneuver). Figure 3A depicts the basic structure of a scene defined by DSO.

Note that while PandaSet and NuScenes are the primary datasets used in this paper, DSO is not constrained by this choice. Rather, DSO is *dataset agnostic* and is designed to describe any driving scene, regardless of its source. In other words, it could just as easily be used to describe scenes originating in all other AD datasets mentioned in Section 3.1. When developing DSO, we included entity types encountered in the NuScenes, Lyft and Pandaset datasets while manually unifying and normalizing the concepts with similar semantic types across the datasets.

In DSO, two types of Scene are represented: SequenceScene and FrameScene. SequenceScene represents the situation in which an ego-vehicle drives over a interval of time and along a path of spatial locations; often captured as video. FrameScene represents the situation of an ego-vehicle at a specific instant of time and point in space; often captured as an image, and generated by sampling the frames of a video. A FrameScene may be a part of a SequenceScene if its time instant and spatial point are *within* the time interval and spatial path of the SequenceScene, respectively (Figure 3B).

Time may be represented in several ways. Firstly, each FrameScene is annotated with a time instant, encoded as xsd:dateTime. Each SequenceScene is annotated with two time instants, representing the beginning and end of a time interval. Secondly, scenes may be linked to other scenes based on their relative temporal order, using the relations occursBefore and occursAfter. Spatial information is linked to a Scene through the hasLocation property. The range of hasLocation is a SpatialRegion, which may be expressed as a Geometry (in GeoSPARQL) (Perry and Herring, 2012) with latitude and longitude coordinates or (inclusive) as an Address with country, province, city, street, etc.

An Entity is a perceived object or event, and is linked to a Scene (i.e. either FrameScene or SequenceScene) through the includes relation. The Entity class is divided into two subclasses, Object and Event. An Object may participate in an Event, represented with the is Participant Of and has Participant relations. 38 classes are defined as a subclass of Entity (either as an Object or Event); derived from the 3D bounding box annotation labels and semantic segmentation annotation labels used by PandaSet (Section 3.1). Table 2 lists the primary relations associated with a Scene.

**TABLE 2:** Relations associated with a Scene, including their domain and range.

Relation	Domain	Range
*beginTime*	SequenceScene	xsd:dateTime
*endTime*	SequenceScene	xsd:dateTime
*hasLocation*	Scene	SpatialRegion
*hasPart*	SequenceScene	FrameScene
*hasParticipant*	Event	Object
*hasTime*	FrameScene	xsd:dateTime
*Includes*	Scene	Entity
*isParticipantOf*	Object	Event
*isPartOf*	FrameScene	SequenceScene
*occursAfter*	Scene	Scene
*occursBefore*	Scene	Scene

The Driving Scene Knowledge Graphs (DSKG) are generated by converting the scene data contained in each AD dataset (Section 3.1) to RDF^2^ format (Lassila et al., 1998), conformant with the Driving Scene Ontology. The PandaSet SDK^3^, and NuScenes SDK^4^ are used to query and extract the relevant scene data from each dataset, making this process trivially straightforward. The RDF is then generated using the RDFLib^5^ Python library (version: 4.2.2). The resultant KG from Pandaset (DSKG-P) contains 3.3M triples and 53K entities whereas NuScenes KG contains 5.9M triples and 2.11M entities. To make NuScenes KG more scalable for subsequent experiments, we create a sampled version (DSKG-N) by selecting frames in a sequence at every 4 s instead of every 0.5 s in the original KG. Note that entities are instantiated and related only to the FrameScene in which they occur. Table 3 shows the basic statistics of these KGs.

**TABLE 3:** Basic KG statistics of Pandaset (DSKG-P) and NuScenes, sampled at 4s (DSKG-N).

	Pandaset	NuScenes (sampled)
# Triples	3,301,929	819,084
# Entity Classes	38	31
# Relations	19	14
# Sequence scene inst.	103	850
# Frame scene inst.	8,240	4,498
# Entity inst.	53,248	277,287
Triples per entity ratio	62.01	2.95
Avg. cardinality of entity class	15.7	9.07
Avg. cardinality of entity inst.	369.6	57.78

**FIGURE 4:**
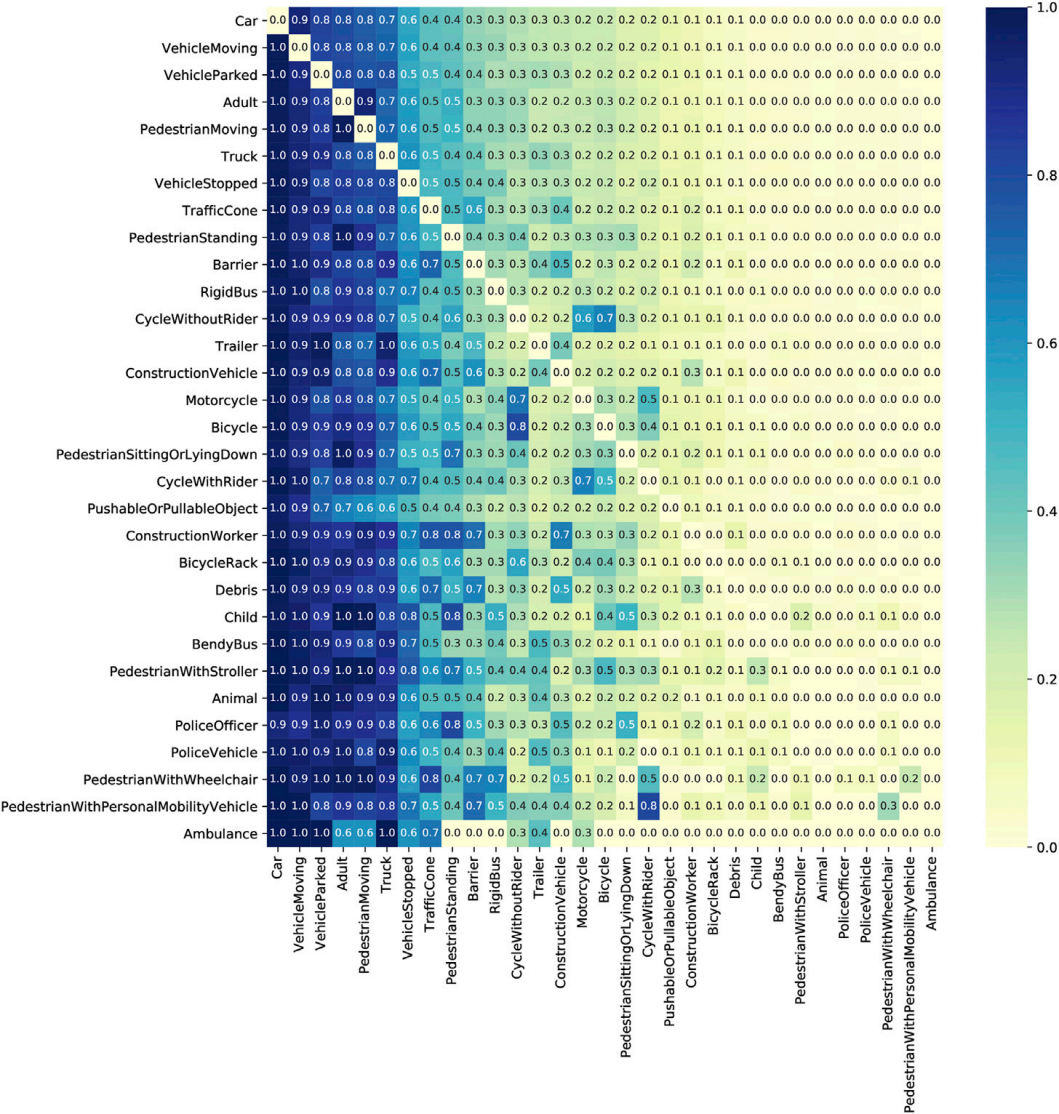
Co-occurrence of entity types within scenes in DSKG-N. Each cell value represents the frequency of Frames in which two entities co-occur, normalized row-wise by the total frequency of Frames in which the row entity occurs.

By analyzing the entity co-occurrences in driving scenes, we find that some entity classes—e.g. moving vehicles, parked vehicles, pedestrians—co-occur with high frequency in urban driving scenes, while some classes—e.g. ambulances, pedestians with wheelchairs—seldom co-occur with other classes (Figure 4). Further, to obtain a relative measure of how often two items co-occur with respect to one’s appearance across all frames, we normalize the co-occurrences row-wise in Figure 4 by dividing each cell value from the total frequency of row label. This reveals the asymmetric relationships between two concepts appearing in the dataset. For example, the frequency of seeing a Child in scenes with an Adult in NuScenes is not as same as the frequency of seeing an Adult in scenes with a Child.

## 4 Methodology

**FIGURE 5:**
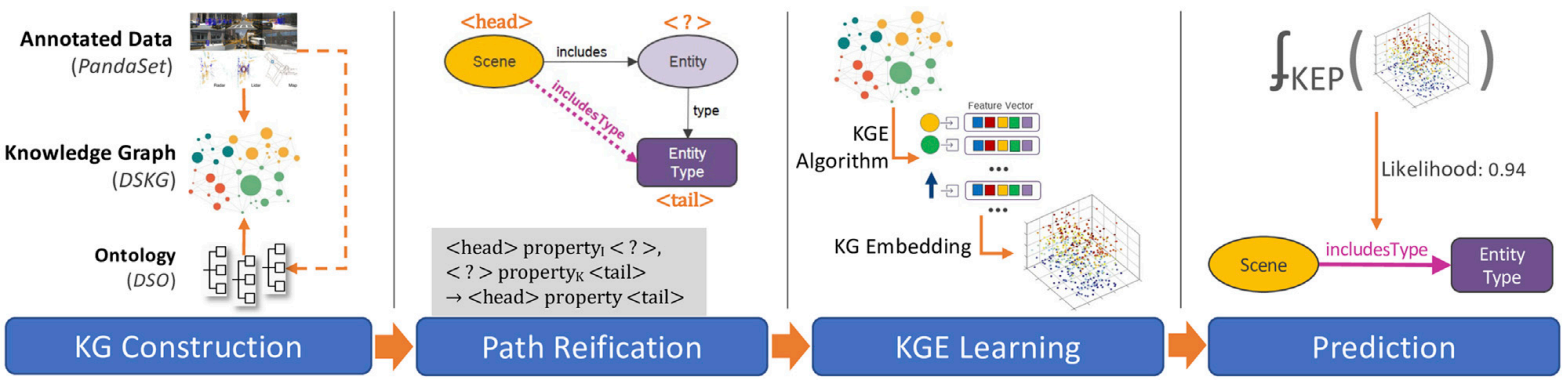
Four main phases involved in Scene Entity Prediction (KEP) pipeline.

The pipeline architecture developed for KEP contains four primary phases, illustrated in Figure 5; including 1) KG construction, 2) path reification, 3) KGE learning, and 4) entity prediction. In this section the final three phases of the architecture are detailed, starting with a scene knowledge graph and ending with a prediction of entities in the scene. First, we formally describe the mapping of the KEP task into a LP problem (Section 4.1). The challenges associated with this mapping are then outlined and addressed (Section 4.2). Next, we describe the process of learning KGEs, along with discussion about the selection of algorithms, algorithmic details and practical challenges (Section 4.3). Finally, we show how entities are predicted using the KGEs (Section 4.4).

In addition, we also include the technical details of two related investigations. The first investigates several alternative KG structures for DSKG in order to understand their relative effect on KEP (Section 6.1). The second investigates the integration of relevant external knowledge of the scene and its effect on KEP (Section 6.2).

### 4.1 Mapping Knowledge-Based Entity Prediction into a Link Prediction Problem

DSKG contains triples of the form ⟨scene_*i*_, includes, car_*j*_⟩ representing an entity instance (car_*j*_) included in a scene (scene_*i*_); Figure 1. Note that entity instances are expressed with all lowercase letters (e.g. car_*j*_ while their corresponding entity classes in title case (e.g. Car). An entity instance is linked to its class in DSO through triples of the form ⟨car_*j*_, rdf:type, Car⟩. In this context, it may be tempting to formulate KEP as a LP problem (described in Section 2.3) with the objective to complete triples of the form ⟨scene_*i*_, includes, ?⟩. This formulation, however, would entail predicting a specific entity *instance* rather than predicting the *class* of an entity. Similar to CV-based object recognition, the objective of KEP should be to predict the **class** of an entity in the scene—e.g. predicting Car rather than *car*_*j*_. In other words, most LP models are unable to complete the triple ⟨*h*, *r*, *t*⟩ when there is no *r* that directly links *h* and *t* in the training data, even if *h* and *t* are linked through a path of *n*-hops (*n* > 1) in the KG, such as: ⟨*h*, *r*_1_, *t*_1_⟩, ⟨*t*_1_, *r*_2_, *t*⟩. This is precisely the issue faced by KEP with the DSKG, as a Scene instance is connected to an Entity sub-class only *via* a 2-hop path. Due to this requirement, KEP cannot simply rely on LP in a straightforward manner. In the next section, we present an approach to overcome this limitation.

### 4.2 Path Reification

As described in Section 4.1, a solution for KEP would require finding the class of an entity. Since class information is not immediately available through a direct link from scene_*i*_, the KEP task may be more accurately formulated as a **path prediction** problem—i.e. predicting the path from a scene instance to a sub-class of Entity. Any solution for KEP should specifically address the path prediction requirement. To overcome this issue, we introduce a new relation to the base DSKG that reifies this 2-hop path. More specifically, the includesType relation directly links Scene instances with Entity sub-classes. This requirement can be more formally defined as follows:

Let s_*i*_ be the *i* ^*th*^ scene instance node in DSKG (s_*i*_ ∈S ), *e*_*j*_ be the *j* ^*th*^ entity instance node ( $e_{j}\in I$ ) and ? be a sub-class of Entity in DSO (? ∈ *E* where *E* = {Car, Animal, Pedestrian, … } ⊆C ). Then:

⟨s_ *i* _, includes, e_ *j* _⟩∧⟨e_ *j* _, rdf:type, ?⟩⇒⟨s_ *i* _, includesType, ?⟩

$$\left\langle {\text{s}}_{i},includes,{\text{e}}_{j}\right\rangle \wedge \left\langle e_{j},rdfs:type,?\right\rangle \Rightarrow \left\langle {\text{s}}_{i},includesType,?\right\rangle$$ (1)

With this addition, DSKG is transformed into DSKG_*R*_; i.e. DSKG with reified paths. Since the includesType relation is now present during training, it will enable the re-use of LP methods. As a result, KEP can now be mapped to LP in order to complete triples of the form ⟨s_*i*_, includesType, ?⟩ in DSKG_*R*_. As an added advantage, this transformation also allows KEP to be used in predicting the type of instances that can possibly be new/non-existent/missing at the time of KG creation.

### 4.3 Transforming KGs to KG Embeddings

The KGE learning with LP objective results in generating a latent space that may be useful for many downstream applications [e.g. (Celebi et al., 2019; Mohamed et al., 2020)] and various other tasks such as querying, entity typing, and semantic clustering (Jain et al., 2021). For these reasons, our approach for KEP involves learning KGEs using several KGE algorithms and re-using the learned latent space for KEP. For this task, our KGE algorithm selection strategy is two-fold: 1) select one *popular* (Jia et al., 2020) representative algorithm from each of the three classes mentioned in Section 2.3, and 2) select algorithms with space and time complexities lower than $O\left(n^{2}\right)$ , efficient enough to conduct multiple experiments. Considering these criteria, 3 KGE algorithms are selected for experimentation: TransE, HolE, and ConvKB.

First, TransE (Bordes et al., 2013), one of the most popular and representative KGE model, learns relations between nodes as a geometric translation in the embedding space. This, however, limits it’s ability to handle symmetric/transitive relations, *1-to-N* relations and *N-to-1* relations (Rossi et al., 2021). Second, HolE (Nickel et al., 2016) uses the circular correlation (denoted by ⋆ in Table 4) among head and tail of a triple with its relation embedding to learn an efficient compression of a *full expressive* (Kazemi and Poole, 2018) bi-linear model. This allows both nodes and relations to be represented in $R^{d}$ . Finally, ConvKB (Nguyen D. Q. et al., 2018) learns a high-level feature map of the input triple by passing a concatenated node/relation embeddings through a convolution layer with Ω set of filters (# filters *τ* = |Ω|). The fact score is then computed by using a dense layer with only one neuron and weights *W*. Table 4 summarizes the scoring function of each algorithm along with their space and time complexities.

**TABLE 4:** Details of selected KGE algorithms: class of algorithm, triple scoring functions and their space and time complexities. **Notation used**: m=|N| , n=|R| , *n*_*t*_ = # of training triples, $h,r,t\in R^{d}$ , *τ* = |Ω| = # of convolution filters.

Algorithm	Class	Scoring function	Space (S) and time (T) complexity
TransE	Geometric	*f*_*r*_ (*h*, *t*) = − |**h** + **r** − **t**|_1/2_	**S:** O(md+nd) **T:** $O\left(n_{t}d\right)$
HolE	Matrix factorization	$\left[h⋆t\right]=\sum_{k=0}^{d-1}{\left[h\right]}_{k}.{\left[t\right]}_{\left(k+i\right)\mathrm{mod}d}$ *f*_*r*_ (*h*, *t*) = **r** ^*T*^ (**h** ⋆ **t**)	**S:** O(md+nd) **T:** $O\left(n_{t}d\log d\right)$
ConvKB	Deep learning	*f*_*r*_ (*h*, *t*) = *concat* (*g* ([**h**, **r**, **t**]∗Ω)).*W*	**S:** O(md+nd+(τ+3)d) **T:** $O\left(n_{t}\tau d\right)$

### 4.4 Entity Prediction Using Knowledge Graph Embeddings

To use KGEs for KEP, we first learn an embedding space from DSKG using the three selected algorithms. Notably, there are a few key differences between KEP and the traditional LP setup. The KGE algorithms for LP learn to maximize the estimated plausibility *ϕ* (*h*, *r*, *t*) for any valid triple while minimizing it for any invalid, or negative, triple. Such KGE models can then be used to infer any missing link by obtaining the element (*h*? or *t*?) with the highest plausibility to complete the triple ⟨*h*, *r*, *t*⟩. In contrast, the objective of KEP is to predict a *specific link* captured by triples of the form: ⟨s_*i*_, *includesType*, ?⟩. To enable this more specific link prediction, a KGE representation of nodes and relations are first learned using the LP objective. Then, for each scene $s_{i}$ , the KGE is queried using includesType relation to find the missing *k* entity class labels $L_{k}\subseteq E$ (see line 5–10 in Algorithm 1). Note that, for the experiments presented in this paper, we consider scene $s_{i}$ to be an instance of FrameScene. However, depending on the application of KEP, $s_{i}$ can be either a FrameScene or SequenceScene. In the case of a SequenceScene, all entities included in each FrameScene, within a sequence, could be aggregated and linked directly to the SequenceScene $s_{i}$ . Algorithm 1 succinctly describes the proposed KEP process, given a KGE model trained using any KGE algorithm. The computational complexity of the proposed algorithm is O(N×M) where N=|S| and M=|E| .

Algorithm 1 Knowledge-based Entity Prediction (KEP) algorithm

## 5 Evaluation

In this section, a detailed evaluation of KEP is conducted. First, the evaluation setup and metrics considered for KEP are introduced. The performance of KEP is then evaluated on each dataset considering the complete DSKG with path reification (*DSKG*_*R*_). Second, an association rule-mining approach is introduced as a baseline for comparison and its performance is evaluated against the KEP approach.

**Table:** 

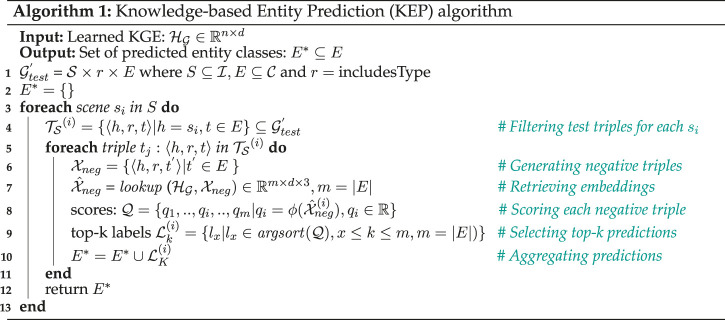

### 5.1 Evaluation Setup

The first step of each KEP experiment is to train KGEs using the three selected algorithms. The training phase is not different from the traditional LP setup. To ensure consistency, we use the algorithm implementations^6^ provided by the Ampligraph library (version 1.3.1) (Costabello et al., 2019). Considering tunable hyper-parameters, the embedding dimension (*k*) is set to 100 across all algorithms and with batch count of 100. Due to the high cardinality of entity instances per scene, the generation of negative triples is restricted to five for each positive triple. We use the multi-class negative log-likelihood (Multiclass-NLL) loss function proposed by (Toutanova and Chen, 2015) where both the head and the tail of triples are corrupted to generate negatives. This loss is then minimized during training using Adam (Kingma and Ba, 2014) as the optimizer. To prepare the datasets for evaluation, each KG is divided into train, validation and test subsets with an 8:1:1 ratio while also ensuring that there are no unseen entities present in the valid/test sets. Additionally, when evaluating the performance of KEP, we filter the test subset to include only triples with the includesType relation. All experiments are performed on a system with Intel Xeon Platinum 8260 CPU @2.40 GHz and NVIDIA TESLA V100 GPU (32 GB GPU memory).

During the evaluation, the learned embedding model is queried to complete triples of form ⟨s_*i*_, includesType, ?⟩ (Algorithm 1, line 9)^7^. In contrast to the traditional LP evaluation where candidates for the *tail* of this triple include all nodes in the KG—i.e. ?∈N —in our setup, the *tail* is restricted to only entity sub-class nodes—i.e. ? ∈ *E*.

Several evaluation metrics are used to quantify the performance of KEP. As our evaluation is a special case of traditional LP, we can re-use the metrics common in LP literature. The first group of metrics, referred to as ranking metrics, include: 1) *Mean Reciprocal Rank (MRR)* (Eq. (2)) that captures the average of inverse entity prediction ranks, and 2) *Hits@K* (Eq. (3)) that calculates the proportion of test triples—containing the includesType relation—with an entity prediction rank that is equal or less than a specified threshold (*K*). The range of values for both *MRR* and *Hits@K* are between 0 and 1, with the higher value indicating better model performance. The values reported in this paper for these metrics use “filtered” setting ensuring that none of the corrupted negatives are actually positives.

$$MRR=\frac{1}{\left|Q\right|}\underset{q\in Q}{\sum }\frac{1}{\left|Q\right|}\text{ where}\;Q=\text{set of ranks from test predictions}$$ (2)

$$Hits@K=\frac{\left|q\in Q:q\leq K\right|}{\left|Q\right|}\text{ where}\;Q=\text{set of ranks from test predictions}$$ (3)

The second group of metrics captures the overall KEP task performance. The first metric to consider is KEP accuracy. When the DSKG is divided into train/test subsets, some parts of a scene may be included with the training set while others could be included with the valid or test set. Hence, during testing, the objective is to measure how well the KGE model can recover the unseen entity classes of a scene in the test set. Specifically, given a scene $s_{i}\in S_{test}$ (i.e. set of scenes in the test set), let entity classes missing from $s_{i}$ during testing be $E_{s}^{\left(i\right)}\subseteq E$ , and the predicted entity classes linked to $s_{i}$ be $L_{p}^{\left(i\right)}\subseteq E$ (i.e. *K* highest ranked entities, $K=|E_{s}^{\left(i\right)}|$ ). Note that KEP accuracy is an example-based evaluation metric (i.e. evaluated per-scene) and that $L_{p}^{\left(i\right)}$ disregards entity classes that are present in train and validation sets. The KEP accuracy is defined as:

$$KEP\;Accuracy=\frac{1}{\left|S_{test}\right|}\overset{\left|S_{test}\right|}{\underset{i}{\sum }}\frac{\left|L_{p}^{\left(i\right)}\cap E_{s}^{\left(i\right)}\right|}{\left|E_{s}^{\left(i\right)}\right|}$$ (4)

Next, we consider two metrics widely used in multi-label classification tasks to evaluate the per-label performance of KEP. Even though KEP—unlike traditional multi-label classification—does not predict the full set of labels for a given scene, the evaluation metrics for multi-label classification can still be useful considering the subset of labels predicted at test time. In this regard, label-based metrics can be used to evaluate the performance on each class label separately and then with micro/macro averaging across all classes. For KEP, we consider both macro and micro averaged F1-scores [Eq. (5)]. While *macro-averaging* captures the arithmetic mean of the *per-class* F1 values, *micro-averaging* considers all samples together to compute the (micro-averaged) precision and recall first, and then combine them using Eq. (5). Futher details about these metrics can be found in (Zhang and Zhou, 2014). Note that macro-averaged F1 gives equal weight to each class. Therefore, to evaluate a problem with class imbalance, such as ours, micro-averaged F1 would be a better fit.

$$F1\;score=2.\frac{precision\times recall}{precision+recall}$$ (5)

### 5.2 Association Rule Mining as a Baseline

To our knowledge, there are no existing baselines that provide a direct comparison with the KEP task. In this section we will establish a baseline considering an alternative approach. Recall that the objective of KEP is to predict a subset of (new) labels given a partially observed set of labels. Given this objective, we may ask the question of how would the co-occurrence of labels (i.e. label association) help predict the missing set of labels. Association rule mining (ARM) is an unsupervised data-mining technique that can be used to uncover the associations among different items in a set by considering their co-occurrence frequencies. For example, in retail market basket analysis, ARM is successfully used to find associations among items that a customer frequently buys together. Viewed in this manner, KEP can be formulated as a market basket analysis problem, where the set of basket items represent the set of observed labels in a scene. The rules ARM generates take the form: *r*_*i*_: {*A*, *B*}⇒{*C*}|*c* where the antecedents {*A*, *B*} imply the co-occurrence of consequent {*C*} with a confidence factor of *c*; 0 ≤ *c* ≤ 1, indicating minimum *c%* transactions in the set of transactions *T* satisfying *r*_*i*_ rule. For the entity prediction task, an association rule mining approach contains three primary steps. First, a set of association rules are generated using the Apriori algorithm on the training dataset (Agrawal et al., 1996). Second, for each scene, a mask is created considering the rules whose antecedents are subsets of the observed set of labels in the training set. Finally, the set of predicted labels are obtained by aggregating the unique set of consequents to satisfy the mask created above. The accuracy is calculated by averaging the proportion of test labels correctly predicted for each scene.

### 5.3 Evaluation Results

The KEP evaluation results are presented, including performance on the path reified DSKGs—DSKG-P_*R*_ (Pandaset) and DSKG-N_*R*_ (NuScenes)—along with the ARM baseline results. Table 5(A) summarizes the results of the ARM baseline and Table 5(B,C) show the KEP results on DSKG-P_*R*_, DSKG-N_*R*_, respectively. When considering evaluation on DSKG-P_*R*_, both ranking metrics (i.e. MRR, Hits@K) and KEP performance metrics (i.e. accuracy, macro/micro-averaged F1) across all three algorithms, HolE performs significantly better than ConvKB and TransE. On the contrary, ConvKB and TransE perform better compared to HolE on DSKG-N_*R*_. When considering the two datasets, KEP peak performance is significantly higher with Pandaset (88.91% compared to 36.35%). The association rule mining baseline achieved average accuracy of 27.19%, which is significantly lower than the peak accuracy obtained using HolE on Pandaset (88.91%), however, still 9.36% better than ConvKB’s inferior performance.

**TABLE 5:** KEP results of association rule mining (ARM) baseline (A), DSKG_*R*_ generated using Pandaset (DSKG-P_*R*_) and NuScenes (DSKG-N_*R*_) on three algorithms, each experiment averaged with standard deviation across five runs (B,C), followed by the results of the additional investigations: different KG structures (D,E) and integration of external knowledge (F). Evaluation metrics: MRR = Mean Reciprocal Rank, H@K= Hits@K, Accu. = KEP Accuracy, Micro/Macro F1 = Micro/Macro-averaged-F1-score.

			Ranking metrics				KEP performance metrics		
			MRR	H@1	H@3	H@10	Accu. (%)	Micro F1	Macro F1
(A)	ARM	—	—	—	—	—	27.19	0.16	0.06
(B)	DSKG-P_*R*_	TransE	0.32 ± 0.03	0.16 ± 0.05	0.35 ± 0.04	0.71 ± 0.03	22.98 ± 4.33	0.26 ± 0.04	0.20 ± 0.02
		HolE	**0.93 ± 0.00**	**0.87 ± 0.01**	**0.98 ± 0.00**	**1.00 ± 0.00**	**88.91 ± 0.64**	**0.90 ± 0.01**	**0.87 ± 0.00**
		ConvKB	0.29 ± 0.01	0.11 ± 0.02	0.31 ± 0.02	0.86 ± 0.02	17.83 ± 1.99	0.22 ± 0.02	0.17 ± 0.02
(C)	DSKG-N_*R*_	TransE	0.42 ± 0.03	0.22 ± 0.03	0.51 ± 0.03	0.91 ± 0.01	28.08 ± 2.45	0.32 ± 0.03	0.20 ± 0.01
		HolE	0.23 ± 0.01	0.11 ± 0.01	0.22 ± 0.01	0.51 ± 0.03	13.80 ± 0.84	0.16 ± 0.01	0.11 ± 0.01
		ConvKB	**0.49 ± 0.02**	**0.31 ± 0.04**	**0.60 ± 0.02**	**0.91 ± 0.01**	**36.35 ± 2.96**	**0.40 ± 0.03**	**0.20 ± 0.01**
(D)	DSKG_*Bi*_	TransE	0.41	0.19	0.52	0.97	29.03	0.34	0.32
		HolE	0.29	0.11	0.28	0.87	16.55	0.19	0.20
		ConvKB	0.23	0.07	0.21	0.68	12.30	0.16	0.14
(E)	DSKG_*Prot*_	TransE	0.26	0.10	0.28	0.62	17.77	0.21	0.18
		HolE	0.33	0.17	0.32	0.81	23.70	0.27	0.22
		ConvKB	0.30	0.10	0.36	0.86	19.21	0.24	0.20
(F)	DSKG_SE_	TransE	0.30	0.18	0.32	0.50	24.53	0.27	0.17
		HolE	0.81	0.69	0.92	0.98	74.52	0.82	0.81
		ConvKB	0.29	0.13	0.32	0.71	21.01	0.26	0.22

“Bold” values in (B, C) indicate the peak performance for each metric in DSKG-R, while “underlined” values in (D,E, and F) indicate the same for each additional investigation.

## 6 Additional Investigations

Our proposed solution for KEP motivated an investigation of two other incidental issues. First, we setup experiments to investigate the effect of various KG structures on the KEP task. Second, a preliminary evaluation is conducted to examine the effect of integrating external knowledge of scenes from OSM (*DSKG*_SE_). Note that Pandaset is used as the dataset to conduct all additional investigation.

### 6.1 Investigation into Different Knowledge Graph Structures

**FIGURE 6:**
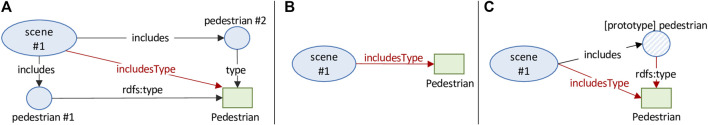
Different KG structures: **(A)** DSKG_*R*_: KG with all instances + reified paths, **(B)** DSKG_*Bi*_: KG with only reified paths, **(C)**: DSKG_*Prot*_: KG with reified and prototype paths.

In DSKG-P, each scene is linked to a high number of entity instances, resulting in a high cardinality of the includes relation. This situation results in a large KG, and thus the KGE training process can be time consuming with poor scalability. This situation motivates a question related to the structure of DSKG: Would an alternative, more compact, representation of a scene yield better performance? To answer, we consider three different graph patterns (Figure 6), including the *DSKG*_*R*_ discussed previously, and compare their performance on the KEP task:

1) Complete Graph (DSKG_*R*_)—All entity instances and entity types are linked to the scene (Figure 6A).
2) Bipartite Graph (DSKG_*Bi*_): Only entity types are linked to the scene (Figure 6B).
3) Prototype Graph (DSKG_*Prot*_): Entity types are linked to the scene along with a single prototype instance for each distinct entity type (i.e. the prototype represents all entity instances of this type) (Figure 6C).

Each pattern represents entity instance information along the path from a scene to an entity class in a slightly different way. DSKG_*R*_ is the KG described throughout this paper and provides the most expressive representation of a scene including all entity instances and classes, along with the added includesType relations. DSKG_*Bi*_ is a more compact representation and contains only the includesType relations between scenes and entity types, discarding all the entity instances and includes relations from the graph. This pattern results in a bipartite-graph structure linking scenes and entity types. The resulting entity instance cardinality for each scene is reduced to zero while maintaining the same entity class cardinality. DSKG_*Prot*_ is similar to DSKG_*Bi*_, but instead of removing all entity instances, they are replaced with a single prototype instance for each linked entity class. Note that this prototype instance represents all the entity instances of a particular entity class that are linked to a scene. In this case, the resulting entity instance cardinality for a scene is equal to the entity class cardinality.

#### Results for Investigation into Different Knowledge Graph Structures

The KEP results are presented when the alternative KG structures, DSKG_*Bi*_ and DSKG_*Prot*_, are used. The entity prediction results using the bipartite graph structure (DSKG_*Bi*_) are significantly poor as compared to the results with DSKG_*R*_, with the use of TransE as an exception (Table 5D).

Now we look at how KEP performs using the DSKG with prototype instances. Note that this KG version contains some information about entity instance nodes, as opposed to DSKG_*Bi*_, but at a minimal level when compared with DSKG_*R*_. The results are summarized in Table 5E and show that this minimal entity instance information may be useful for outperforming DSKG_*Bi*_, but it still underperforms the complete DSKG_*R*_.

### 6.2 Investigation into Integrating External Knowledge

A key advantage of representing scenes in a KG is that it allows for the integration of information from external sources. This begs the question of whether integrating additional knowledge about a scene would enhance the KEP performance. To demonstrate the process and test the hypothesis, we incorporate additional location attributes that enrich the spatial semantics of scenes. The underlying dataset, Pandaset, records GPS coordinates (i.e latitude and longitude) for each frame. Since the numeric representation of latitude/longitude does not carry much semantic information about the location, we enriched each frame with location attributes queried from Open Street Map (OSM)^8^. This process is two-fold: First, a reverse query of the latitude/longitude is executed using OSM Nominatim^9^, which returns relevant address information such as the City, County, StreetName, etc. This information is added to an Address instance that is created and linked to the scene (Figure 3A). Second, OSM-tags are leveraged to find additional entities in the scene, such as ParkingLane, Highway, Building, etc. These additional entities are instantiated and linked to the scene instance via the includes relation. Compared to DSKG_*R*_, the resultant KG, termed *DSKG*_SE_, contains 34.14% additional entity classes and 3.19% additional triples.

#### Results for Investigation into Integrating External Knowledge

We evaluate the performance of entity prediction when the DSKG_*R*_ is enriched with external information from OSM, resulting in DSKG_SE_. As shown in Table 5F, the enrichment from OSM did not yield better overall predictive performance across all three algorithms. However, it did slightly improve the performance of TransE and ConvKB when the prediction conditions are tougher with Hits@1 and KEP accuracy. When it comes to the best performing algorithm, HolE, the OSM enrichment actually hinders its performance.

## 7 Analysis and Discussion

The evaluation of KEP above leads to some interesting observations. First, when considering the hypothesis tested in the paper—i.e. *whether an expressive KG structure used within a knowledge-infused learning approach could help predict the unrecognized entities in a scene*—our evaluation suggests that the unrecognized entities can indeed be predicted with high precision when the DSKG is constructed from a high-quality dataset (e.g., 0.87 Hits@1 and 88.91% accuracy in Pandaset). Next, we’ll discuss other findings of our evaluation considering 4 aspects: 1) dataset perspective, 2) algorithmic perspective, 3) investigation into KG structure and the importance of instance information, 4) integration of external knowledge.

First, when considering the two datasets used for experiments, the results clearly show the superior performance on Pandaset compared to NuScenes. While this could be due to several reasons, we divulge into two possible reasons: 1) diversity of content in scenes, and 2) differences in dataset (and resultant KG) structure. When considering the increased diversity of content in scenes, NuScenes has a richer diversity than Pandaset. For example it includes scenes from cities in two continents (Singapore and Boston), different driving patterns (left/right-hand driving), and variety of weather/traffic conditions. Next, when considering the dataset structure, different design decisions impacted the overall structure of the datasets and the resultant KGs. Specifically, each dataset handles objects occurring across frames differently. Pandaset keeps the same identifier for an object across frames while NuScenes introduces unique identifier for an object in each frame. For NuScenes this results in a very large entity space (2.11M in original KG, 277K in the sampled version) and a sparse KG (e.g., entity instance cardinality is 6.4 times less in NuScenes compared to Pandaset). Additionally, the triples per entity ratio is significantly less in NuScenes (2.95 compared to 62.01 in Pandaset, Table 3), making the KGE learning task difficult due to lack of training triples about entities. Therefore, the differences in diversity and dataset structure could lead to a more challenging prediction task for NuScenes. It is important to note that the quality of the KG is heavily dependant of the quality of the underlying data, in terms of annotation quality and coverage, which impacts the performance of KEP. This evaluation highlights the challenge and importance of creating, selecting, and/or cleaning a dataset that is suitable for the kind of approach presented in this paper.

Second, when considering the KGE algorithms used for evaluation, these results clearly show the superior performance of HolE on the KEP task. This may be a consequence of HolE’s ability to handle graph patterns with higher instance cardinality, as it can represent 1-to-N, N-to-1 and N-to-N relations through circular correlation (⋆) (see scoring function in Table 4). TransE, however, lacks this ability to represent such relations and ConvKB suffers from the same as it can be considered as a DL-based extension of TransE (Jia et al., 2020).

**FIGURE 7:**
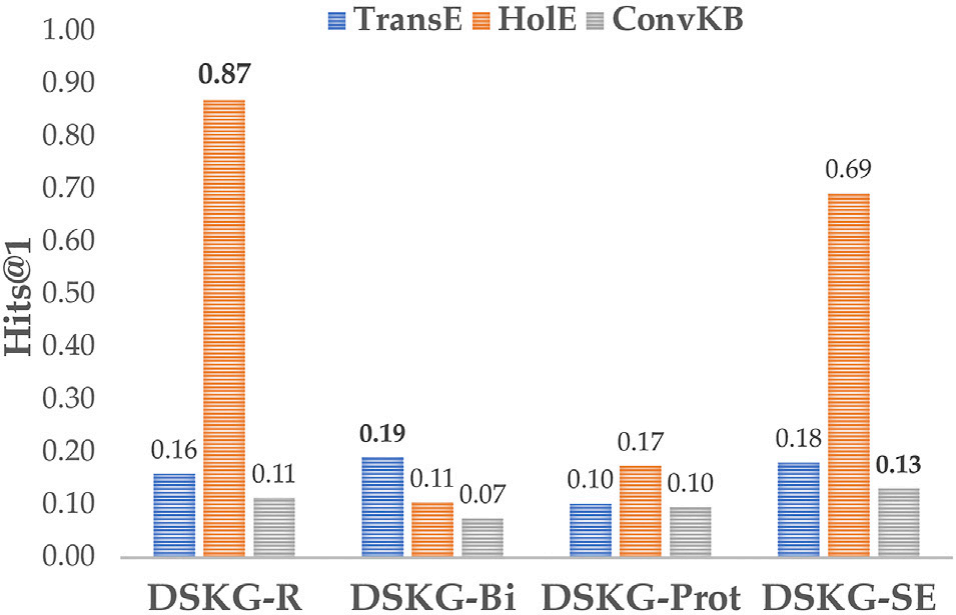
Hits@1 variation of KGE algorithms over different KG structures.

Third, the investigation into the use of different KG structures indicates that entity instance information along the path from scenes to entity classes may be important even when the prediction task does not consider this information directly (recall Figure 6). Figure 7 shows that the Hits@1 performance with HolE increases with an increasing number of paths (DSKG_*R*_>DSKG_*Prot*_>DSKG_*Bi*_). Having more of these paths, and entity instances, directly increases the number of 1-to-N relations associated with a scene. HolE can better capture such relations, leading to better KEP performance.

Fourth, our investigation into integrating external knowledge from OSM shed some light on a practical issue with knowledge integration. Even though this integration did slightly help the poorly performing TransE and ConvKB, it negatively impacted the best performing algorithm—HolE. One potential explanation could be that the contribution of only 3.19% new triples from the enrichment is hugely disproportionate to the 34.13% increase in label space. Hence, the newly added triples do not provide enough new training data to support the added complexity of the prediction task.

Finally, we will shed some light into the generalizability of this approach for other domains and problems. With the current approach, the path to be predicted, and subsequently reified, is required to be known apriori. Therefore, the approach presented in the paper can be generalizable to any problem that naturally fits this constraint.

## 8 Conclusions and Future Work

This paper defines an innovative process for entity prediction that leverages relational knowledge of driving scenes. The limitations of LP methods are explored and ultimately overcome through path reification. Our evaluation justifies the hypothesis tested in the paper by suggesting that unrecognized entities can be predicted with high precision of 0.87 with the HolE KGE algorithm. We believe this approach is generalizable to a range of problems and use-cases, both within AD and beyond, which is the focus of future work. In addition, we’d also like to explore the benefits of an end-to-end framework with joint learning of embeddings. The evaluation and analysis has led to many interesting open and challenging research questions to be explored in future work, including 1) how to leverage temporal relations among Frames in a Sequence to improve KEP, 2) how to transfer knowledge from one dataset/KG to another in order to perform KEP, and 3) deriving effective mechanisms to integrate and leverage external knowledge of the scene. Nontheless, it’s clear that knowledge-infused learning is a potent tool that may be effectively utilized to enhance scene understanding for autonomous driving systems.

## Data Availability

The original contributions presented in the study are included in the article/Supplementary Material, further inquiries can be directed to the corresponding author.

## Footnotes

1
https://scale.com/open-datasets/pandaset#overview

2
https://www.w3.org/RDF/

3
https://github.com/scaleapi/pandaset-devkit

4
https://github.com/nutonomy/nuscenes-devkit

5
https://github.com/RDFLib/rdflib

6
https://docs.ampligraph.org/en/1.3.1/ampligraph.latent_features.html

7
https://docs.ampligraph.org/en/latest/generated/ampligraph.discovery.query_topn.html

8
https://www.openstreetmap.org/

9
https://wiki.openstreetmap.org/wiki/Nominatim
